# Repeat IGRA Testing in Canadian Health Workers: Conversions or Unexplained Variability?

**DOI:** 10.1371/journal.pone.0054748

**Published:** 2013-01-31

**Authors:** Alice Zwerling, Andrea Benedetti, Mihaela Cojocariu, Fiona McIntosh, Filomena Pietrangelo, Marcel A. Behr, Kevin Schwartzman, Dick Menzies, Madhukar Pai

**Affiliations:** 1 Department of Epidemiology, Biostatistics & Occupational Health, McGill University, Montreal, Canada; 2 Respiratory Epidemiology & Clinical Research Unit, Montreal Chest Institute, Montreal, Canada; 3 Department of Medicine, McGill University Health Centre, Montreal, Canada; 4 Department of Occupational Health & Safety, McGill University Health Center, Montreal, Canada; 5 Research Institute of the McGill University Health Center, Montreal, Canada; National Institute for Infectious Diseases (L. Spallanzani), Italy

## Abstract

**Background:**

Although North American hospitals are switching from tuberculin testing (TST) to interferon-gamma release assays (IGRAs), data are limited on the association between occupational exposure and serial QuantiFERON-TB Gold In-Tube (QFT) results in healthcare workers (HCWs).

**Methods:**

In a cohort of Canadian HCWs, TST and QFT were performed at study enrolment (TST1 and QFT1) and 1 year later (TST2 and QFT2). Conversion and reversion rates were estimated, and correlation with TB exposure was assessed.

**Results:**

Among 258 HCWs, median age was 36.8 years, 188/258 (73%) were female and 183/258 (71%) were Canadian-born. In 245 subjects with a negative QFT1 we found a QFT conversion rate of 5.3% (13/245, 95% CI 2.9–8.9%). Using more stringent definitions, QFT conversion rates ranged from 2.0 to 5.3%. No TST conversions were found among the 241 HCWs with negative TST1, and no measure of recent TB exposure was associated with QFT conversions. In the 13 HCWs with a positive QFT1, 62% reverted.

**Conclusion:**

Using the conventional QFT conversion definition, we found a higher than expected rate of conversion. Recent occupational exposures were not associated with QFT conversions, and no TST conversions occurred in this cohort, suggesting the ‘conversions’ may not reflect new TB infection.

## Introduction

Interferon-gamma release assays (IGRAs) are increasingly being used for the diagnosis of latent TB infection (LTBI) instead of the tuberculin skin test (TST) [1], [2], [3]. IGRAs offer certain logistical advantages over the TST and have been shown to have at least comparable sensitivity and improved specificity in BCG vaccinated individuals [4], [5], [6]. However, early research into serial testing with IGRAs has suggested there may be problems in the implementation of these tests for repeat screening, and interesting case studies have emerged from US hospitals that have switched in recent years to QuantiFERON-TB Gold In-Tube (QFT) for HCW screening [2], [3], [7], [8], [9], [10].

In particular, there are concerns IGRAs will have lower rates of initial positivity than the TST (due in part to improved specificity) but later result in higher numbers of conversions in serial testing settings, leading to more HCWs being considered for LTBI therapy. Traditionally, an increase in annual TST conversion rate is a marker of potential transmission in the hospital and would signal that institutional infection control measures needed to be improved [11], [12], [13], [14], [15]. Yet it remains unclear whether higher conversion rates reported from studies using IGRAs truly reflect nosocomial transmission or a hospital outbreak. Indeed, many health care institutions in the United States that switched to IGRAs have found much higher QFT conversion rates than expected with the TST - up to 5 times higher than expected [8], [10], [16]. However, to date there have been no other indications of increased TB transmission at these sites, and consequently no change in infection control practices.

IGRA reversions in the absence of treatment have also been noted, leading to a concern over unnecessary treatment of low risk health care workers. Evaluating LTBI diagnostics is further complicated by a lack of gold standard for diagnosing latent TB. Therefore we must ask whether the increased rate of QFT conversions are indeed true conversions, or simply reflect either an underlying dynamic immunologic process, problems with test reproducibility or even random within-person variation.

The use of IGRAs for serial testing is complicated further by lack of data on optimal cut-offs for serial testing, and unclear interpretation and prognosis of conversions and reversions [17], [18]. In a systematic review of studies on short term reproducibility of IGRAs, all 4 of the studies identified reported significant within-person IGRA variability, and more recent studies have confirmed this finding [2], [19], [20], [21], [22].

Given these concerns, we need to understand how IGRAs perform in serial testing programs for HCWs, and whether conversions are indeed associated with TB exposure. Current Canadian IGRA guidelines do not recommend use of IGRAs for serial testing HCWs [23], [24]. To inform future updates, we conducted a serial testing study among HCWs in Montreal.

### Study Objectives

Our primary study objective was to assess changes in QFT results over one year in a cohort of HCWs in low TB risk hospitals, and to assess whether QFT conversions and reversions were associated with occupational exposure, treatment for LTBI or other known TB risk factors. We were also interested in determining whether alternative conversion definitions were more strongly associated with occupational TB exposure and risk factors.

## Methods

### Setting & Population

This cohort study was conducted at the McGill University Health Centre (MUHC), in Montreal. Methods and results from the cross-sectional analysis of this cohort at study enrolment have been published elsewhere [25]. The MUHC comprises a network of hospitals, employing more than 11,000 health care and support personnel. TB incidence on the Island of Montreal is estimated at 7.1 per 100,000 persons [26]. Most of the HCWs included in this cohort worked at the Montreal General Hospital (MGH), the Royal Victoria Hospital (RVH), or the Montreal Chest Institute (MCI). In 2011, 34 cases of TB were diagnosed at the MCI and 8 at the MGH, therefore classifying both as moderate to high risk hospitals for TB exposure [15]. Two cases of TB were diagnosed in the same year at the RVH, making it a low risk hospital.

HCWs at the MUHC are required to undergo screening for LTBI both pre-employment and as part of regular occupational screening. Current practice for LTBI screening at the MUHC uses the TST, and is conducted by the Department of Occupational Health and Safety (OHS). HCWs are required to undergo TST at the time of employment, and annual TST after that, except for high-risk HCWs (e.g. TB clinic and TB laboratory staff) who are required to undergo TST every 6 months [27]. Based on studies conducted among HCWs in Montreal, the annual risk of TB infection (ARI) among HCWs in low TB risk hospitals was estimated to be under 1%, and among moderate and high TB risk hospitals, the estimated ARI was around 2.7% [28], [29]. However, these studies were conducted over a decade ago. Since then, TB incidence in Montreal (and Canada) has steadily decreased as has the annual number of TB admissions in the MUHC hospital system [28], [30], [31].

All participants provided written informed consent and the study received approval from the research ethics board of the McGill University Health Centre. HCWs were approached for consent during their regular pre-employment or annual appointment with OHS. Participants with a previous positive TST (≥10 mm) or those who had received a TST within the last 6 months were excluded. Consistent with the Canadian guidelines, LTBI treatment decisions were based only on TST results [27].

At enrolment, QFT1 and TST1 were performed. Blood was drawn directly into pre-coated QFT tubes. For the TST, 5TU (0.1 ml) of Tubersol PPD (Aventis Pasteur) was administered using the Mantoux method. After 48–72 hours, the transverse diameter was demarcated using the ballpoint pen method, measured and recorded in millimetres by a trained research nurse as per the Canadian TB Standards [27]. The QFT assay was performed as per the manufacturer’s instructions. Participants were contacted again 1 year after study enrolment for QFT2 and TST2. TST2 was not performed in participants with a positive TST1. QFT2 was performed in all participants regardless of QFT1 results, including those with positive TST1.

Interviewer facilitated questionnaires were administered at study enrolment and again at 1 year follow-up testing At enrolment, the questionnaire covered occupational and non-occupational TB exposure prior to enrolment, while the questionnaire at 1 year covered TB exposure since TST1 and QFT1. Known unprotected occupational exposure to TB was assessed both by self-recall, through cross-checking lists of occupational TB exposures provided by OHS and through chart reviews of all HCWs’ OHS dossiers. Key occupational exposures included: working in a high risk location, non-occupational exposure to TB (social, school, family, etc), and known unprotected occupational exposure to TB patient. High risk work locations were identified as work in the emergency department, intensive care unit, infectious disease unit, immunodeficiency services, respiratory therapy, microbiology lab, TB clinic, TB research (with patient contact), TB laboratories or the AIDS clinic.

### Test Procedures and Criteria for Conversions and Patterns of Change

A two-step baseline TST was performed, as per the Canadian TB standards for TST1 on all newly employed HCWs or any HCW who had not received TST in the past 10 years [27]. A TST conversion was defined as an induration of <10 mm at baseline, and a TST2 induration of 10 mm or greater, with an absolute increase of ≥10 mm over TST1 [32].

Quantitative values of interferon-gamma (IFN-g) were recorded. As per the manufacturer’s suggestion, all IFN-g values greater than 10 were truncated at 10 IU/ml, and negative values were re-scaled to 0. QFT conversions were defined in five different ways, based on previous literature:

- A: change from a negative QFT1 (IFN-g <0.35 IU/ml) to a positive result for QFT2 (≥0.35 IU/ml) (Source: manufacturer’s definition; also included in the US CDC IGRA guideline [33])
- B: change from negative QFT1 to QFT2≥0.35 IU/ml AND at least 2.6 times QFT1 value (Source: preliminary serial testing results from large US study of HCWs [34])
- C: change from negative QFT1 to QFT2 at least 0.7 IU/ml greater than QFT1 (Source: within-subject variability measured in South African study [35] )
- D: change from negative QFT1 to QFT2≥1.0 IU/ml (Source: analysis of QFT1 results of this cohort [25])
- E: change from negative QFT1 to QFT2≥1.6 IU/ml (Source: preliminary serial testing results from large US study of HCWs [34])

QFT reversions were defined as a change from positive QFT1 result (≥0.35 IU/ml) to a negative QFT2 result at 1 year follow-up (IFN-g <0.35 IU/ml).

Patterns of change in dichotomous QFT results were defined as: *stable negatives:* QFT1 and QFT2 both negative, *stable positives:* QFT1 and QFT2 both positive, *converters:* QFT1 negative and QFT2 positive, and *reverters:* QFT1 positive and QFT2 negative. For these definitions, the manufacturers’ recommendations for cut-offs were used.

### Statistical Analysis

All analyses were performed with Stata, Version 11 (Stata Corp, Texas, USA). Conversion rates were estimated for each definition and Fisher’s exact 95% confidence intervals (CI) were estimated. To determine which QFT conversion definition was most closely associated with indicators of occupational exposure to TB, we fit 5 multivariable logistic regression models using each of the 5 different QFT conversion definitions as the outcome. The Mann-Whitney test was used to assess whether there was a statistically significant difference in median IFN-g values at test 1 across different temporal patterns of QFT (ie: stable positives versus reverters).

Participants (n = 4) with indeterminate results at either study enrolment (time 1) or 1 year follow-up (time 2) were excluded from the above regression models.

## Results

The participant characteristics of our HCW cohort at enrolment were described in a previous publication [25]. Of the 388 HCWs who completed TST1 and QFT1, 258 completed all tests at TST2 and QFT2, between 2007 and 2012. Characteristics of the 258 participants included in this analysis are presented in Table 1. Their median age was 36.8 years (IQR:27.2–45.9 years), 188/258 (73%) were female, 183/258 (71%) were Canadian born and 99/258 (38%) were BCG vaccinated. 88/258 HCWs (34%) were working in a hospital with less than 6 cases of active TB admissions/year (classified as low risk for TB exposure), while the remaining 170/258 (65.9%) HCWs were working in moderate to high risk facilities with more than 6 cases of active TB/year.

**Table 1:** Participant characteristics, n = 258 (at study enrolment unless otherwise stated).

Participant Characteristics	N (%)
AgeMedian, IQR	36.8 yrs (27.2–45.9 yrs)
SexFemaleMale	188 (72.9%)70 (27.1%)
Country of birthCanadian born (non-aboriginal)Foreign born: low TB inc. (≤25/100, 000)Foreign born: moderate TB inc (26–100/100,000)Foreign born: high TB inc (>100/100,000)	183 (70.9%)43 (16.7%)15 (5.8%)17 (6.6%)
Educational levelHigh school or lessCollege or university degreePost graduate degree	34 (13.2%)163 (63.2%)61 (23.6%)
Job categoryNon- clinical staffNursing staffMedical doctorsOther clinical staffLaboratory staff	83 (32.2%)57 (22.1%)23 (8.9%)80 (31.0%)15 (5.8%)
BCG vaccinationNo vaccinationAt birth or within 1 yearPost infancyReceived multiple BCG vaccinationsUnknown timing	159 (61.6%)41 (15.9%)22 (8.5%)6 (2.3%)30 (11.6%)
Direct contact with a patient with TB between time 1 and time 2YesNo	27 (10.5%)231 (85.5%
Non-occupational TB exposure between time 1 and time 2NoYes	257 (99.6%)1 (0.4%)
Total years worked in health careMedian, IQR	6 yrs (2–15yrs)
Worked in a high risk area for TB exposure between time 1 and time 2NoYes	243 (94.2%)15 (5.9%)
Prior TSTYes – negativeYes – but not readNoDon’t know	222 (86.1%)7 (2.7%)26 (10.1%)3 (1.2%)

While non-occupational exposure between enrolment and 1 year follow-up testing was a rare event (1/258, 0.4%), documented occupational exposure to TB between testing was reported by 27/258 HCWs (10.5%). HCWs who did not complete TST2 and QFT2 were similar to those who completed the study in terms of age, sex, job type, country of birth, education, and BCG vaccination status. No HCWs were diagnosed with active TB during the study period, however 3 HCWs in this cohort completed LTBI therapy post study enrolment, all for positive TST1 results.

### TST Conversions

Among the 258 HCWs who completed testing, 17 participants had positive TST1 results and TST2 was therefore not performed. Among the 241 HCWs who underwent TST2, only one TST value above 10 mm was reported. This HCW had a TST1 result of 6 mm and a TST2 result of 15 mm, which did not meet the pre-specified criteria for TST conversion. Therefore, there were no TST conversions in this cohort.

### QFT Conversions and Reversions

Using the manufacturer’s definition of change from negative to positive (IFN-g ≥0.35 IU/ml) we estimated a QFT conversion rate of 5.3% (13/245, 95% CI: 2.9–8.9%). As seen in Table 2, conversion definition B identified the same number of conversions as the manufacturer’s definition (A). Conversion definitions C and D (absolute increase of 0.7 IU/ml above QFT1, and ≥1.0 IU/ml respectively), both estimated QFT conversion rates of 2.5% (6/245, 95% CI: 0.9–5.3%). Using the most strict conversion definition E: ≥1.6I U/ml, we estimated a 2% QFT conversion rate (5/245, 95% CI: 0.7–4.7%).

**Table 2:** QFT Conversion rates using manufacturer’s recommended and alternative conversion definitions.

QFT Conversion Definition from negative QFT1	n/N (%)	95% Confidence Interval
A: change from negative to positive (≥0.35 IU/ml)	13/245 (5.3%)	2.9–8.9%
B: QFT2 IFN-g ≥0.35 IU/ml & absolute increase of 2.6 times above QFT1 IFN-g value	13/245 (5.3%)	2.9–8.9%
C: QFT2 IFN-g ≥0.35 IU/ml & absolute increase of 0.7IU/ml above QFT1 IFN-g value	6/245 (2.5%)	0.9–5.3%
D: change from negative QFT1 to QFT2 IFN-g ≥1.00 IU/ml	6/245 (2.5%)	0.9–5.3%
E: change from negative QFT1 to QFT2 IFN-g ≥1.6 IU/ml	5/245 (2.0%)	0.7–4.7%

QFT =  QuantiFERON-TB Gold In Tube.

IFN-g = interferon-gamma.

IU = International Units.

QFT reversions were seen in 8/13 (61.5%) HCWs with positive QFT1 results. All 8 were negative on TST1 and TST2. Half of these reverters had QFT1 positive results ranging from 1.00–10 IU/ml, while the remaining 4 were between the cut-point value of 0.35 IU/ml and 1.0 IU/ml (0.39–0.74). Of the 6 with QFT1 values between 0.35 and 1.0 IU/ml, 4/6 (66.7%) reverted at QFT2, while 4/7 (57.1%) of those with QFT1 values over 1.0 IU/ml reverted at QFT2. None of the reverters had received treatment for LTBI.

### QFT Conversion and Occupational Exposure

We assessed TB exposure prior to TST1 and QFT1, and TB exposure between TST1/QFT1 and TST2/QFT2. We hypothesized that if QFT conversions represent new instances of TB infection they should be associated with recent TB exposure (ie: exposure between QFT1 and QFT2). However, no QFT conversions, using any conversion definition, were found among any of the 26 QFT1 negative HCWs who reported recent occupational TB exposure. Similarly, no conversions were seen among the 14 HCWs who reported being moved to a high risk work location between QFT1 and QFT2 or any of the 16 HCWs born in countries with high TB incidence (ie. >100 cases per 100,000 persons) (Table 3). Characteristics of the 13 HCWs with QFT conversions are presented in Table 4.

**Table 3:** Occupational factors associated with QFT Conversion (using manufacturer’s conversion definition).

	Participants with QFTConversion (%)	Participants with noQFT conversion
Job CategoryNon-clinical staff*DoctorsNursesClinical staffLaboratory staff	6 (7.2%)3 (13%)2 (3.5%)1 (1.3%)1 (6.7%)	7720557914
Non-Occupational TB Exposure prior to study enrolmentNo reported exposureSelf-reported TB exposure	11 (4.5%)2 (14.3%)	23312
Travel outside Canada >1 month prior to enrolmentNoYes	5 (2.6%)8 (12.3%)	18857
Worked as a HCW in a foreign countryNoYes	8 (3.4%)5 (19.2%)	22421
Known unprotected occupational exposure to active TBNoYes	11 (5.5%)2 (3.5%)	19055
Country of BirthLow TB IncidenceModerate TB IncidenceHigh TB Incidence	12 (5.3%)1 (6.7%) 0 (0%)	2141417
Self reported occupational exposure between time 1 and time 2 †NoYes	13 (5.6%)0 (0%)	21827
Changed work location to high risk between time 1 and time 2NoYes	13 (5.3%)0 (0%)	23015
Diagnosed with LTBI between time 1 and time 2NoYes	13 (5.1%)0 (0%)	2405
Completed treatment for LTBI between time 1 and time 2NoYes	13 (5.1%)0 (0%)	2423

† time 1 was study enrollment and includes TST1, QFT1 and questionnaire, time 2 was 1 year after study enrollment, and included TST2, QFT2 and a questionnaire on exposure between time 1 and time 2.

**Table 4:** Characteristics of 13 participants with QFT conversions during study.

Participant	TST 1 Result (mm)	TST 2 Result (mm)	QFT 1 Result (IU/ml)	QFT 2 Result (IU/ml)	% of time with patients	Total Years working in health care	BCG Vaccinated	HCW in a foreign country	Job Category
1	0	0	0.1	10	76–100%	10	No	Yes	Nurse
2	0	0	0	0.38	76–100%	12	Yes (8 yrs old)	No	Clinical
3	0	3	0.04	10	26–50%	10	No	No	Housekeeping
4	0	0	0	1.6	0%	12	No	No	Laboratory (Not microbiology)
5	0	0	0.24	1.17	1–25%	20	Yes (5 yrs old)	No	Warehouse staff
6	0	0	0.07	0.56	76–100%	11	No	No	Nurse
7	0	0	0.03	0.7	0%	5	Yes- timing unknown	Yes	Research
8	0	0	0	0.48	1–25%	1	Yes (5 years old)	No	Clinical
9	8	0	0	0.65	0%	13	No	No	Administration
10	0	0	−0.01	0.64	0%	1	No	Yes	Administration (High TB risk hospital)
11	10	–	0.19	0.51	0%	10	Yes (1 year old)	Yes	Medical doctor
12	0	0	0.03	2.53	1–25%	7	No	No	Medical doctor
13	15	–	0.13	2.2	1–25%	17	Yes (At birth)	Yes	Medical doctor

Using logistic regression we assessed which occupational and TB risk factors, if any were associated with QFT conversions (using conventional and alternative definitions). However, the samples sizes were quite small with only 5 or 6 outcomes for alternative conversion definitions. There was no association between recent TB exposure and QFT conversions using any definition (data not shown). All significant variables (having worked as a HCW in a foreign country, total years having worked in health care, and non-occupational TB exposure) were all exposures that occurred prior to QFT1.

### QFT Temporal Patterns

Among the 258 HCWs with complete results for QFT1 and QFT2, we found 228/258 (88.4%) were stable negatives, 5/258 (1.9%) were stable positives, 13/258 (5.0%) were QFT converters and 8/258 (3.1%) were QFT reverters. Table 5 displays QFT results at 1 year stratified by QFT1 result.

**Table 5:** Dichotomous QFT results at time 1 and time 2.

QFT1	QFT2
244/258 (94.6%) QFT (−) Negative	228/244 (93.4%) Negative
	13/244 (5.3%) Positive
	3/244 (1.2%) Indeterminate
13/258 (5.0%) QFT (+) Positive	8/13 (61.5%) Negative
	5/13 (38.5%) Positive
1/258 (0.4%) QFT Indeterminate	1/1 (100%) Negative

QFT =  QuantiFERON-TB Gold In Tube.

TST = tuberculin skin test.

When QFT patterns were stratified by TST1, HCWs with positive TST1 results had higher rates of stable QFT positive values (23.1%) and converters (15.4%), compared with TST1 negative HCWs with a 0.8% rate of stable positives and 4.5% converters. When we assessed QFT patterns stratified by TST1/QFT1 discordance, (Table 6), we found a much higher rate of reversion among those with discordant TST1/QFT1 (80%) results compared to those with concordant positive TST1/QFT1 results (0%).

**Table 6:** 1 year QFT pattern stratified by TST1 and QFT1 results.

TST 1 & QFT 1	1 year QFT pattern (%)
Concordant negatives (n = 234)	3 Indeterminates (1.3%)
	220 Stable Negatives (94%)
	**11 Converters (4.7%)**
Discordant (TST+/QFT−) (n = 10)	8 Stable Negatives (80%)
	**2 Converters (20%)**
Concordant positives (n = 3)	3 Stable Positives (100%)
	0 Reverters
Discordant (TST−/QFT+) (n = 10)	2 Stable Positives (20%)
	**8 Reverters (80%)**
TST−/QFT Indeterminate (n = 1)	1 QFT Negative

QFT =  QuantiFERON-TB Gold In Tube.

TST = tuberculin skin test.

### Change in Interferon-gamma Responses between QFT 1 and QFT 2

**Figure 1:**
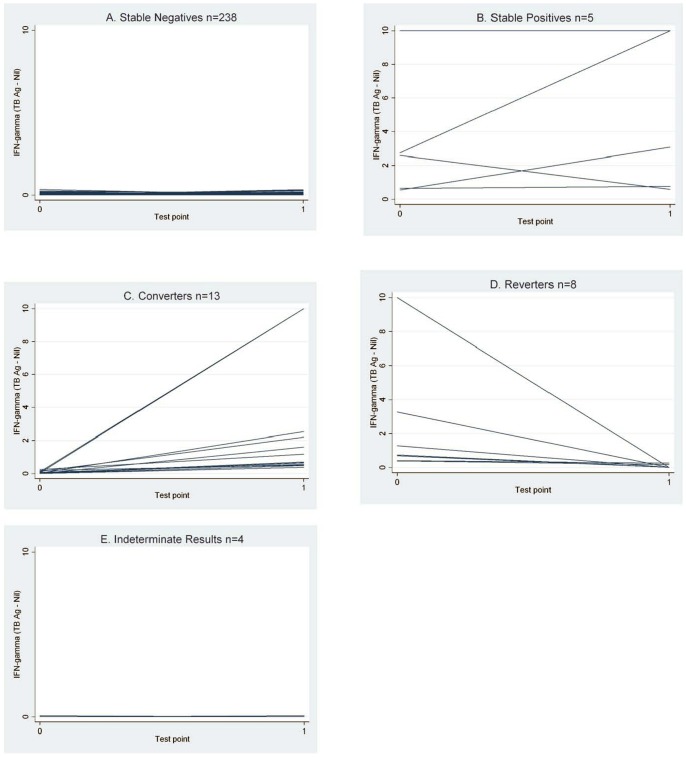
Trajectories of continuous IFN-gamma values from time 1 to time 2. Trajectory of continuous interferon-gamma response over time within each participant (TBAntigen- TB Nil, IU/ml). HCWs have been stratified by overall QFT patterns and we have presented trajectory plots for each category of QFT pattern: stable negatives, stable positives, converters, reverters, and indeterminate results.

Trajectory plots of continuous INF-g measurement from the QFT1 and QFT2, stratified by QFT pattern are presented in Figure 1: A thru E. When subdivided into QFT patterns, the trajectories are similar: negatives show little variability, while converters and reverters have IFN-g values ranging from 0–10 IU/ml. Given the cut-off of 0.35 IU/ml, and that negative values have been truncated at 0, there is thus little possibility for the negatives to show much variability in IFN-g response over time.

We calculated the difference in IFN-g levels between QFT2 and QFT1. Unlike the distribution of IFN-g itself, change in IFN-g had a relatively normal distribution. Table 7 displays the median and inter-quartile ranges (IQR), along with mean and standard deviations (SD) stratified by QFT pattern. As expected, the biggest change in INF-g values is seen among converters (median change in IFN-g = 0.67IU/ml) and reverters (median change in IFN-g = −1.01 IU/ml). Stable negatives experienced a median value of 0 change while stable positives reported a small median increase (0.13 IU/ml).

**Table 7:** Median and IQR values for QFT1 and difference in Interferon-gamma levels (IFN-g at QFT2– IFNg at QFT1 (IU/ml) and stratified by 1 year QFT pattern.

1 year QFT Pattern	Median change in IFN-gamma, (IQR)	Mean change in IFN-gamma, (SD)	Median IFN-gamma at QFT1 (IQR)	p value for Mann- Whitney rank sum test
Stable Negatives	0 (−0.01, 0.02)	0.01 (0.07)	0 (0, 0.03)	
Stable Positives	0.13 (0.00, 2.53)	1.58 (3.56)	2.59 (0.64, 2.74)	
Converters	0.67 (0.49, 2.07)	2.53 (3.43)	0.03 (0, 0.1)	p = 0.08*
Reverters	−1.01 (−6.64, −0.45)	−3.29 (4.25)	1.01 (0.55, 6.64)	p = 0.88**

* p value for two sample Mann-Whitney rank sum test comparing median IFN-gamma value at QFT 1 between stable negatives and converters.

** p value for two sample Mann-Whitney rank sum test comparing median IFN-gamma value at QFT 1 between stable positives and reverters.

### Interferon Gamma Values Close to the Cut-off and Likelihood of Reversions

Reverters experienced the largest change in IFN-g measured between the two test points. It has been hypothesized that IFN-g values close to the cut-point of 0.35 IU/ml are more likely to revert [2], [7], [8], [9], [36], [37]. In Table 7 we present median IFN-g values for QFT1 stratified by QFT pattern. Here we can see that the median QFT1 IFN-g among reverters was much smaller than that of stable positives (1.01 vs. 2.59 IU/ml), and the IQR was much wider for the reverters compared with stable positives. However when this was assessed using a nonparametric Mann-Whitney test, this difference was not statistically significant.

## Discussion

TB incidence in North America has declined steadily and most Canadian health care workers are currently at low risk for TB exposure [30], [38], [39]. In this context, using conventional QFT conversion definitions, we found a substantially higher than expected annual conversion rate of 5.3%. This is particularly high given the absence of any TST conversions in the same cohort. QFT conversions were not associated with any indicator of recent infection or exposure; suggesting that QFT conversions identified were not true new instances of new TB infection, but may represent unexplained variability in QFT results among this cohort of HCWs.

In the 1990 s, Menzies and colleagues estimated annual TST conversion rates of <1 to 2.7% among HCWs at low and high TB risk hospitals in Montreal [28]. More recently, in a systematic review in 2007, Menzies et al. found a median annual LTBI incidence of 1.1% (range: 0.2 to 12%) for HCWs in high income countries (estimated with TST) [40]. Currently, we would expect a TST conversion rate of about 1% in Canadian HCWs and this is much lower than QFT conversion rates estimated in the present study. We explored alternative QFT conversion definitions, and as expected, more stringent definitions yielded lower estimated conversion rates (2 to 2.5% compared with 5.3%), but it remains unclear whether these reflect real cases of new infection in this low incidence and low TB risk setting.

Many studies have shown that IGRAs are dynamic tests with large within-subject variability over time [2], [7], [8], [13], [20], [21], [22], [35], [36], [37]. One possible explanation for the high QFT conversion rate is that some conversions may be false positive results at QFT2 while others were false negatives at QFT1. While exposures prior to enrolment were correlated with QFT conversions, none of the QFT conversion definitions were associated with occupational exposures between QFT1 and QFT2, leading us to wonder if these are indeed true cases of new infection? We found no TST conversions in this cohort; if we expect a 1% conversion rate, it is possible that our sample size was simply too small to detect such a low TST conversion rate.

An alternative explanation may be that QFT conversions were not the result of recent exposure or newly acquired LTBI but a response to some earlier stimuli. While the QFT test itself cannot cause boosting, it is possible the TST1 could cause a “boosting” effect that is picked up by the QFT2. Exposures associated with QFT conversions were all pre-enrolment exposures including: total years worked as a HCW, having worked as a HCW in a foreign country and non-occupational exposure to TB prior to TST1/QFT1 [25]. This may indicate the evidence of a boosting “effect”. To date most research into this area suggests we would expect the boosting effect to wane after a period of weeks or months, but it can occur after 1 year [16], [41]. In addition, the exposure associated with QFT conversions could have served to sensitize these HCWs, making them more prone to boosting.

While pre-enrollment exposure to TB included any lifetime exposure prior to study enrollment, we cannot eliminate the possibility that these exposures occurred immediately prior to TST1/QFT1, and TST and possibly QFT conversions are thought to happen weeks to months after initial TB exposure. It was interesting to note that having worked as a HCW in a foreign country was the only TB risk factor found to be associated with QFT positivity in the cross-sectional analysis published earlier. We did find all participants with TST1/QFT1 concordant positive (TST+/QFT+) results had stable QFT patterns (n = 3); though numbers were small.

We also found different pre-enrolment exposures were associated with different QFT conversion definitions, including having worked as a HCW in a foreign country prior to study enrolment. These results seem to suggest QFT may be associated with variables reflecting cumulative exposure to TB and not recent exposure in this setting. This is in contrast to a popular hypothesis that IGRAs should be better associated with recent exposure and that the TST is better associated with markers of cumulative lifetime exposure to TB.

We found a high QFT reversion rate in our cohort (8/13, 61.5%, 95% CI: 31.6 −86.1%), and this is consistent with reports from other serial testing studies [8], [21], [36], [37]. It is unclear if these reversions represent a natural clearing of infection, or simply “wobble” around the cut-point due to test reproducibility. While clinicians might not want to treat low risk HCWs who might revert, predicting those who are likely to revert is problematic. While median IFN-g values at QFT1 appeared quite different between stable positives and reverters, this difference was not statistically significant. Half of the 8 reverters had strongly positive QFT1 IFN-g values, all above 1.0 IU/ml, two with values of 10 IU/ml. QFT reversions were not associated with treatment and it remains difficult to predict which QFT positive results may later revert to negative in the absence of treatment.

Recent reports of high rates of IGRA conversions and reversions have prompted new research on reproducibility of IGRAs. A large study recently published by Metcalfe et al. found that the normal expected range variability in TB response upon re-testing of the same sample was +/−0.60IU/ml or 14% [22]. Other reproducibility studies suggest that variation in processing and incubation times even within the manufacturer’s recommended timeframes can produce drastically different results, and within-subject inter-laboratory variations can also occur [42], [43]. Every effort was made to reduce this variability in our study, all study procedures were conducted by a trained nurse, and all samples were processed by the same technician in a research laboratory. Our study protocol required stricter timelines, less variation in processing times and increased supervision compared with normal clinical procedures, therefore we would expect the variation to be less than that expected under routine programmatic conditions.

The variability and reproducibility issues that impact HCW serial testing studies is also noticed in studies that have explored the use of IGRAs for treatment monitoring [44], [45], [46].

Overall, we found high rates of QFT conversions and reversions that could not be easily explained in our setting. Our data, therefore, lend support to the current Canadian recommendations which advise against the use of IGRAs for HCW serial testing [23].
